# Different Levels of Expression of the Clock Protein PER and the Glial Marker REPO in Ensheathing and Astrocyte-Like Glia of the Distal Medulla of *Drosophila* Optic Lobe

**DOI:** 10.3389/fphys.2018.00361

**Published:** 2018-04-11

**Authors:** Wojciech Krzeptowski, Lucyna Walkowicz, Alicja Płonczyńska, Jolanta Górska-Andrzejak

**Affiliations:** Department of Cell Biology and Imaging, Institute of Zoology and Biomedical Research, Jagiellonian University, Krakow, Poland

**Keywords:** glial oscillators, PER, REPO, astrocyte-like glia, ensheathing glia, circadian clocks, *Drosophila* visual system

## Abstract

Circadian plasticity of the visual system of *Drosophila melanogaster* depends on functioning of both the neuronal and glial oscillators. The clock function of the former is already quite well-recognized. The latter, however, is much less known and documented. In this study we focus on the glial oscillators that reside in the distal part of the second visual neuropil, medulla (dMnGl), in vicinity of the PIGMENT-DISPERSING FACTOR (PDF) releasing terminals of the circadian clock ventral Lateral Neurons (LNvs). We reveal the heterogeneity of the dMnGl, which express the clock protein PERIOD (PER) and the pan-glial marker REVERSED POLARITY (REPO) at higher (P1) or lower (P2) levels. We show that the cells with stronger expression of PER display also stronger expression of REPO, and that the number of REPO-P1 cells is bigger during the day than during the night. Using a combination of genetic markers and immunofluorescent labeling with anti PER and REPO Abs, we have established that the P1 and P2 cells can be associated with two different types of the dMnGl, the ensheathing (EnGl), and the astrocyte-like glia (ALGl). Surprisingly, the EnGl belong to the P1 cells, whereas the ALGl, previously reported to play the main role in the circadian rhythms, display the characteristics of the P2 cells (express very low level of PER and low level of REPO). Next to the EnGl and ALGl we have also observed another type of cells in the distal medulla that express PER and REPO, although at very low levels. Based on their morphology we have identified them as the T1 interneurons. Our study reveals the complexity of the distal medulla circadian network, which appears to consist of different types of glial and neuronal peripheral clocks, displaying molecular oscillations of higher (EnGl) and lower (ALGl and T1) amplitudes.

## Introduction

Glial cells of *Drosophila* are as diverse as their counterparts in vertebrates, with which they share many morphological and functional features (for review see Freeman and Doherty, 2006). The surface, cortex, and neuropil glia that are further divided into subtypes having distinct structures and associated with specific sets of neurons, reflect the diversity of glial functions (Edwards and Meinertzhagen, 2010; Edwards et al., 2012).

Glial cells that express the so called clock genes are considered to be the peripheral clocks (oscillators) in the fruit fly circadian (~24 h) timing system, comparable to photoreceptors and other sensory neurons (reviewed in Jackson et al., 2015; Zwarts et al., 2015; Chi-Castañeda and Ortega, 2016). The fundamental feature of the clock function that enables regulating day and night cycles of various physiological functions is the rhythmic expression of the clock genes. *Drosophila* glial cells have been known for over a decade to rhythmically express the core genes of the circadian clock, such as *period* (*per*) and *timeless* (*tim*) (Zerr et al., 1990; Ewer et al., 1992; Ng et al., 2011). Recently, the expression of PER, the core repressor in the clock mechanism that blocks CLK/CYC-activated circadian transcription of target genes (Hardin, 2011), have been shown to oscillate in several glial subtypes, in a manner suggestive of the circadian clock functioning in these cells (Long and Giebultowicz, 2018). Studies of mammalian glial cells have likewise demonstrated the presence of functional clocks in astrocytes and microglia, which express e.g., *Per1* and *Per2* in a rhythmic manner (Prolo et al., 2005; Marpegan et al., 2011; Hayashi et al., 2013; Fonken et al., 2015; Brancaccio et al., 2017; Chi-Castañeda and Ortega, 2018). Therefore, the glial cells in both mammals and in *Drosophila* appear to be equipped with the same core components of the clock mechanism as the central clocks.

Apart from the studies on clock genes rhythmic expression in *Drosophila* glia, there are also reports suggesting that the glia participate in circadian regulation of behavioral rhythms, such as rhythmic locomotor behavior (also via physiological regulation of the neuronal circuitry driving these rhythms), and that gliotransmitters are involved in the circadian rhythmicity (Suh and Jackson, 2007; Ng et al., 2011; Ng and Jackson, 2015). Glial cells of *Drosophila* are also known to contribute to the circadian structural plasticity that so far has been reported in the clock and other brain structures of flies and mammals (Górska-Andrzejak, 2013; Bosler et al., 2015; Herrero et al., 2017). The so called epithelial glial cells (EGl) of the first optic neuropil (lamina) of Diptera display daily changes of their volume (Pyza and Górska-Andrzejak, 2004) and the level of expression of the catalytic α-subunit of sodium-potassium pump, the Na^+^/K^+^-ATPase (Górska-Andrzejak et al., 2009). The EGl also affect the rhythm of morphological changes of the L1 and L2 monopolar cells—the postsynaptic partners of the compound eye photoreceptors (Pyza and Górska-Andrzejak, 2004). They also modulate the circadian changes of abundance of the presynaptic protein Bruchpilot in photoreceptor terminals (Górska-Andrzejak et al., 2013). The glial clocks (and glia-to-neuron communication) also actively contribute to circadian remodeling of axonal projections of the clock neurons, the small ventral Lateral Neurons (s-LNvs) that control the rest-activity cycles in *Drosophila* (Fernández et al., 2008; Herrero et al., 2017). The acute disruption of glial cells internal clock abolishes the circadian changes of s-LNv projections (Fernández et al., 2008). In view of the evidence mentioned above, we follow Jackson (2011) and use the term “glial clocks” for the glia expressing the clock genes.

In this study, we focus on PER-expressing glial cells, which are located in the distal part of the second visual neuropil, the medulla (hereafter referred to as the distal medulla neuropil glia, dMnGl), in vicinity of the PIGMENT-DISPERSING FACTOR (PDF) releasing terminals of the circadian clock large Ventral Lateral Neurons (l-Nvs). According to many published categorizations of *Drosophila* glial cells (Awasaki et al., 2008; Doherty et al., 2009; Edwards and Meinertzhagen, 2010; Hartline, 2011; Edwards et al., 2012), the dMnGl contain two morphologically distinct types of glia, the astrocyte-like glia (ALGl) and the ensheathing glia (EnGl). Their cell bodies reside at the border between the neuropil and the cortex of the medulla in the estimated ratio of 1:1 (Kremer et al., 2017), whereas their processes span the medulla neuropil in the way reflecting its columnar organization (Kremer et al., 2017; Richier et al., 2017).

The ALGl extend complex processes of high structural density deep into neuropil (infiltrating the medulla layers of synaptic connections M1-M8). They are closely associated with neuronal synapses, express transporters for neurotransmitter clearance and contain multiple neurotransmitter recycling pathways (Stork et al., 2012; Richier et al., 2017). ALGl reveal enriched expression of genes that are involved in energy metabolism, redox reactions, and vesicle-mediated transport and secretion (Ng et al., 2016). RNAi-mediated knockdown of some of these genes has been shown to trigger changes in the level and/or circadian pattern of activity of flies, or to induce paralysis (Ng et al., 2016).

The EnGl processes, on the other hand, are closely associated with neuronal arborizations. They invade the synaptic neuropil of distal medulla as sparsely branched columnar structures showing characteristic branching pattern in the neuropil layers M3 and M6, where the photoreceptors R7 and R8 terminate, as well as in the serpentine layer, M7 (Kremer et al., 2017; Richier et al., 2017). Like their mammalian counterparts, the microglia, EnGl have been reported to phagocytose neuronal debris after axonal injury and also during normal synaptic growth (Doherty et al., 2009; Stork et al., 2012).

Like most of the *Drosophila* glia (with the exception of the midline glia), the ALGl and EnGl, express the REVERSED POLARITY (REPO), a homeodomain protein required for correct differentiation of glia in the embryonic nervous system, including the visual system (Campbell et al., 1994; Xiong et al., 1994; Halter et al., 1995). This transcriptional activator regulates expression of other genes (Yuasa et al., 2003). It was shown, that REPO controls synaptic growth at the *Drosophila* larval neuromuscular junction (NMJ), through the regulation of *wingless* (*wg*) expression (Kerr et al., 2014).

The main goal of our microscopic study was to provide a detailed characterization of the expression of the core clock protein PER and the glial marker REPO in the dMnGl, focusing on differences between the two distinct subtypes that jointly populate this neuropil, the astrocyte-like, and ensheathing glia. They reside in a very strategic region of projections of the most important neuronal pacemakers (the LNvs) for generating behavioral rhythms (Helfrich-Förster et al., 2007). We reveal their heterogeneity with respect to the levels of PER and REPO and daily fluctuations in the abundance of the latter. We believe that our study may establish the foundation for further work on the type-dependent specificity of the glial clocks, as well as on their significance in functioning and structural plasticity of the distal medulla circadian network.

## Materials and methods

### Fly strains

The following strains of *Drosophila melanogaster* were used: Canton-S (CS), *EAAT1*-Gal4 (w^*^; P{Eaat1-GAL4.R}2), *alrm*-Gal4, *tim-*Gal4, UAS-mCD8-GFP (*y*^1^w^*^; P{UAS-mCD8.mGFP.LG}10b), UAS-S65T-GFP (w^*^; P{UAS-GFP.S65T}Myo31DF^T2^), *Pdf*^0^ (*w*+; *Pdf*^0^). Flies were reared on standard yeast-cornmeal-agar medium, under day/night cycles (LD 12:12; 12 h of light and 12 h of darkness; ZT0-denotes the beginning of the day; ZT12-denotes the beginning of the night), at 25 ± 1°C.

For studies on daily expression of glial marker REPO, males that emerged from pupae were kept for 6 days either in LD (one group) or DL (reversed cycle, second group). Following such entrainment, the flies kept in LD were decapitated 1 h (ZT1) and 4 h (ZT4) after the lights on, whereas the flies kept in DL, were dissected 1 h (ZT13) and 4 h (ZT16) after the lights off. For other experiments, the flies were always decapitated at the beginning of the day, at ZT1.

### Immunolabeling

Following flies immobilization with CO_2_, heads were dissected and fixed in 4% PFA (paraformaldehyde, Sigma-Aldrich) in 0.1M Phosphate Buffer (PB) for 2 h. They were washed in Phosphate Buffer Saline (PBS), cryoprotected (4°C, overnight) in 25% solution of sucrose in 0. 01 M PBS and mounted in Tissue-Tek medium (Cryomatrix, Thermo Scientific) for crysectioning. Cryosections of 20 μm thickness were cut and immunolabeled with primary antibodies as follows: mouse anti-REPO Ab (1:40, Developmental Studies Hybridoma Bank), mouse anti-PDF Ab (1:500, Developmental Studies Hybridoma Bank) rabbit anti-GFP Ab (1:1000, Novus Biologicals), mouse anti-GFP Ab (1:1000, Novus Biologicals), rabbit anti-PER Ab (1:1000, a kind gift of R. Stanewsky), and rabbit anti-*Drosophila melanogaster* MESENCEPHALIC ASTROCYTE-DERIVED NEUROTROPHIC FACTOR or DmMANF Ab (1:500, GenScript, prepared using PolyExpress Premium Antigen-Specific Affinity Purified pAb and tested for specificity on homogenates of *DmMANF*-deficient flies). Goat anti-rabbit and goat anti-mouse Abs conjugated with AlexaFluor488 (1:1000, Molecular Probes Invitrogen) or with Cy3 (1:500, Jackson ImmunoResearch Laboratories) were used as secondary antibodies. After final washing, slides were closed in either Vectashield, or DAPI-containing Vectashield, medium (Vector). In experiments on daily expression of glial marker REPO, heads that were collected at four different ZTs, were processed and immunolabeled in parallel, under the same conditions.

### Image acquisition and analysis

Fluorescently labeled cryosections were examined using a confocal microscope, Zeiss LSM 510 META. For quantitative comparisons of PER and REPO-specific intensities, the same acquisition parameters were applied to images of the same experimental set. Morphometric analyses were performed using ImageJ software (NIH, Bethesda). The level of signal intensity was manually quantified as a Mean Gray Value (further referred as MGV): the sum of gray values in the selected area divided by the number of pixels within that area. For study on daily expression of glial marker REPO, fluorescence intensities of 20–30 REPO-positive nuclei of the dMnGl (in a single image of the optic lobe) was quantified and 10 hemispheres (from 10 individuals) were collected for each ZT group.

### Statistics

Data were analyzed for normality using Shapiro-Wilk W-test. Datasets from two experimental groups were tested for significant differences using Mann-Whitney Test. One-way ANOVA or its non-parametric counterpart-Kruskal-Wallis Test, were used for multiple comparisons. N represents either the number of cells, or the number of optic lobes assayed. Data are expressed as means ± *SD. p*-values <0.05 were considered to be statistically significant (^*^p < 0.05, ^**^p < 0.01, ^***^p < 0.001, ^****^p < 0.0001).

## Results

### Two populations of the dMnGl (P1 and P2) express PER at different levels

**Figure 1:**
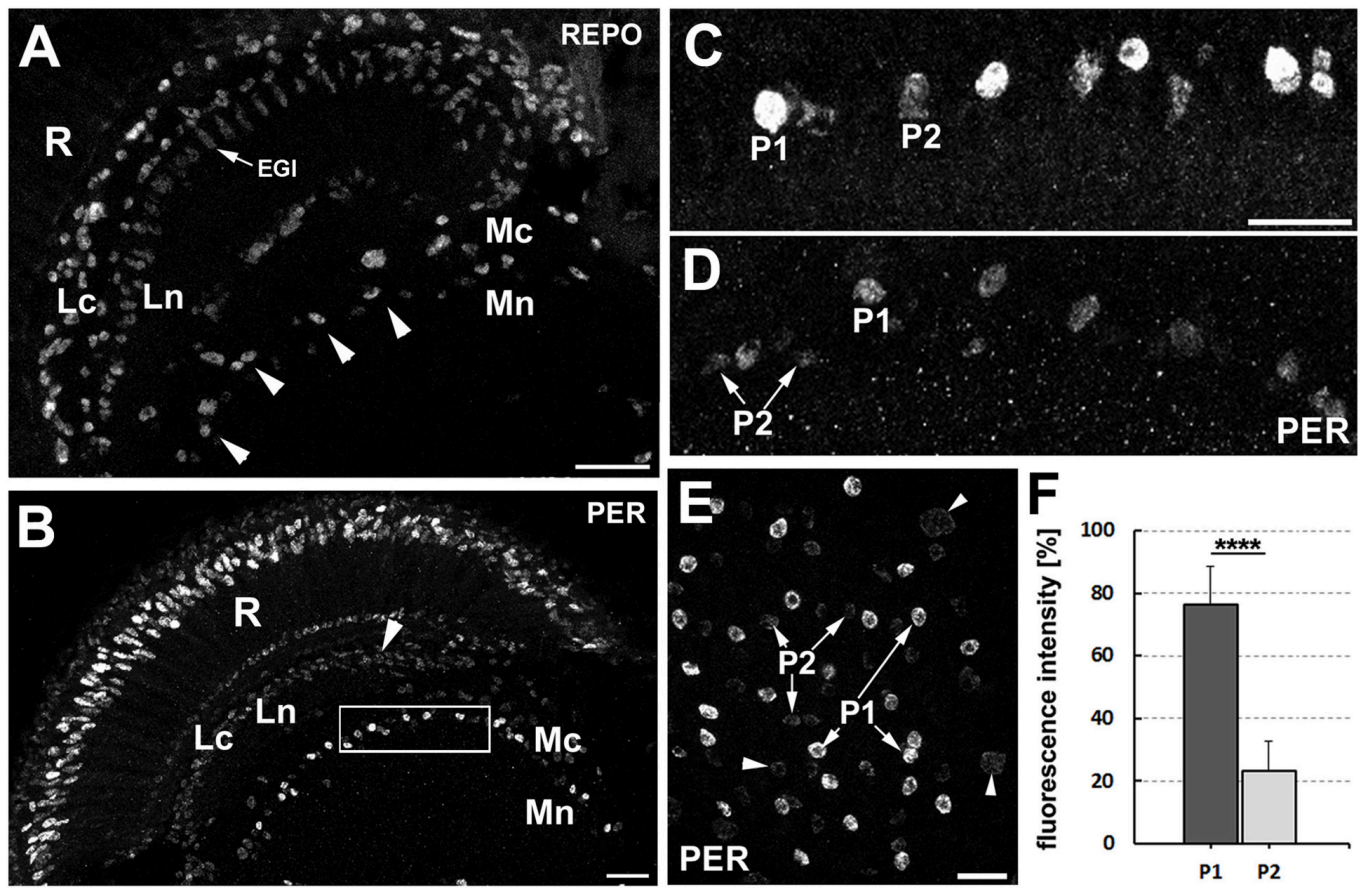
**(A)** Location of the distal medulla neuropil glia (dMnGl) among other glial cells that are marked by REPO-specific immunofluorescence in the optic lobes (frontal section). The nuclei of the dMnGl (arrowheads) reside at the border between the cortex (Mc) and the neuropil (Mn) of the medulla. R, retina; Lc, lamina cortex; Ln, lamina neuropil; EGl, epithelial glial cells. Scale bar: 20 μm. **(B)** Frontal section of *Pdf*^0^ optic lobe immunolabeled with anti-PER antibody. R, retina; Lc, lamina cortex; Ln, lamina neuropil; Mc, medulla cortex; Mn, medulla neuropil. The nuclei of the dMnGl (frame) reveal high level of PER-specific immunofluorescence in comparison with the nuclei of glia in Lc (arrowhead). Scale bar: 20 μm. **(C,D)** Small fragments of the Mc/Mn interface of *Pdf*^0^ **(C)** and CS **(D)** shown in higher magnification reveal the unexpected presence of the dMnGl displaying low level of fluorescence (P2). The P2 cells are well visible in *Pdf*^0^ **(C)**. They are positioned next to cells of high fluorescence (P1) in almost alternating order. Such arrangement can be also observed, although less clearly, in CS **(D)**. The images shown in **(C,D)** were collected at the same image acquisition parameters. Scale bar for **(C,D)**: 10 μm. **(E)** The frontal surface of *Pdf*^0^ medulla. The fluorescence in P2 is comparable to the fluorescence in glial cells of the medulla cortex (arrowheads). Scale bar: 10 μm. **(F)** The average level of fluorescence (±*SD*) in P1 and P2 cells of *Pdf*^0^ (^****^p < 0.0001).

The dMnGl (Figure 1A) display high level of PER-specific immunoreactivity, which is the result of strong PER expression (Figure 1B; Górska-Andrzejak et al., 2018). Closer inspection of the distal medulla has revealed, however, that next to the cells expressing PER at high level (hereafter referred to as P1), there are also cells that express PER at much lower level (hereafter referred to as P2) (Figures 1C,D). They (P2) were initially noticed in *Pdf*^0^ mutant (Figures 1C,E), owing to the relatively high level of PER-specific signal in the glia of this mutant. In *Pdf*^0^ the immunofluorescence in P2 was well visible even though it was 70% weaker than in P1 (Mann-Whitney Test, *Z* = 3.84 [*N*_P1_ = 11 and *N*_P2_ = 10], *p* < 0.0001) (Figure 1F). In CS glia, where the level of PER was generally lower than in *Pdf*^0^, the P2 cells were barely discernible from the background noise (Figure 1D).

### Two populations of the dMnGl (P1 and P2) express REPO at different levels

**Figure 2:**
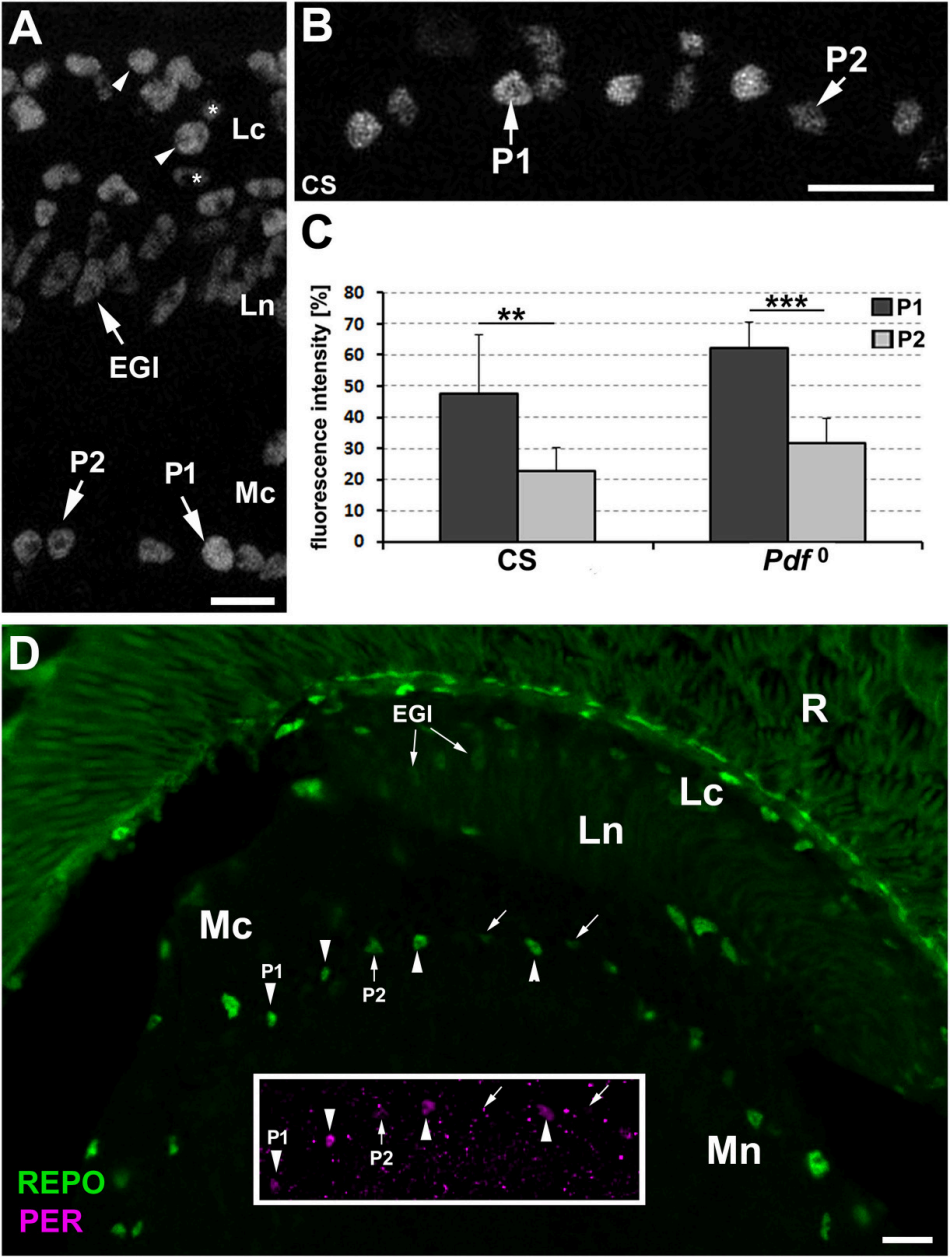
**(A)** The cross section of the lamina and the distal part of the medulla of CS immunolabeled with anti-REPO antibody. The staining reveals differences in the intensity of REPO-specific fluorescence in various types of glia. In the lamina cortex (Lc) some glial cells show higher level of fluorescence (arrowheads) than others (asterisks). Fluorescence of the epithelial glia (EGl) in the lamina neuropil (Ln) is more uniform and rather low. The dMnGl show both higher (P1 cell) and lower (P2 cell) levels of REPO-specific fluorescence. Mc-medulla cortex. Scale bar: 5 μm. **(B)** The P1 and P2 cells of the REPO-positive dMnGl are arranged in almost alternating order. Scale bar: 10 μm. **(C)** The average level of REPO-specific fluorescence (± *SD*) of the P1 and P2 dMnGl in CS and *Pdf*^0^ (^**^p < 0.01, ^***^p < 0.0001). **(D)** The frontal section of the lamina and the distal part of the medulla of CS labeled with anti-REPO and anti-PER (insert) antibodies. The nuclei of REPO-P1 cells (in the main picture, arrowheads) show strong PER-specific fluorescence (insert, arrowheads). The REPO- P2 cells (in the main picture, arrows) reveal weak or no PER-specific fluorescence (insert, arrows). R, retina; Lc, lamina cortex; Ln, lamina neuropil; EGl, the epithelial glia of the lamina; Mc, medulla cortex; Mn, medulla neuropil. Scale bar: 20 μm.

In the next step, we wanted to determine whether P1 and P2 displayed also differences in the level of REPO, the pan-glial marker. This conjecture was brought about by subtle, yet noticeable differences in REPO-specific immunofluorescence observed in different types of the optic lobe glia (Figure 2A), which might reflect some differences in the amplitudes of certain glial functions.

The examination of the distal medulla of CS brains immunolabeled with anti-REPO Ab revealed again the presence of two types of cells that in this case differ in the intensity of REPO-specific fluorescence (Figure 2B). The 52% difference in the intensity of their fluorescence was statistically significant (Mann-Whitney Test, *U* = 6 [*N*_type1_ = 10 and *N*_type2_ = 9], *p* < 0.01). In *Pdf*^0^ mutant we also observed such cells, displaying 49.5% difference in the REPO-specific signal (Mann-Whitney Test, *Z* = 3.93, [*N*_type1_ = 11 and *N*_type2_ = 11], *p* < 0.0001) (Figure 2C). Double immunolabeling (Figure 2D) showed that the cells identified earlier in PER labeling as P1express REPO also at high level, whereas the P2 cells express REPO at low level.

### The number of REPO-P1 cells is bigger during the day than during the night

**Figure 3:**
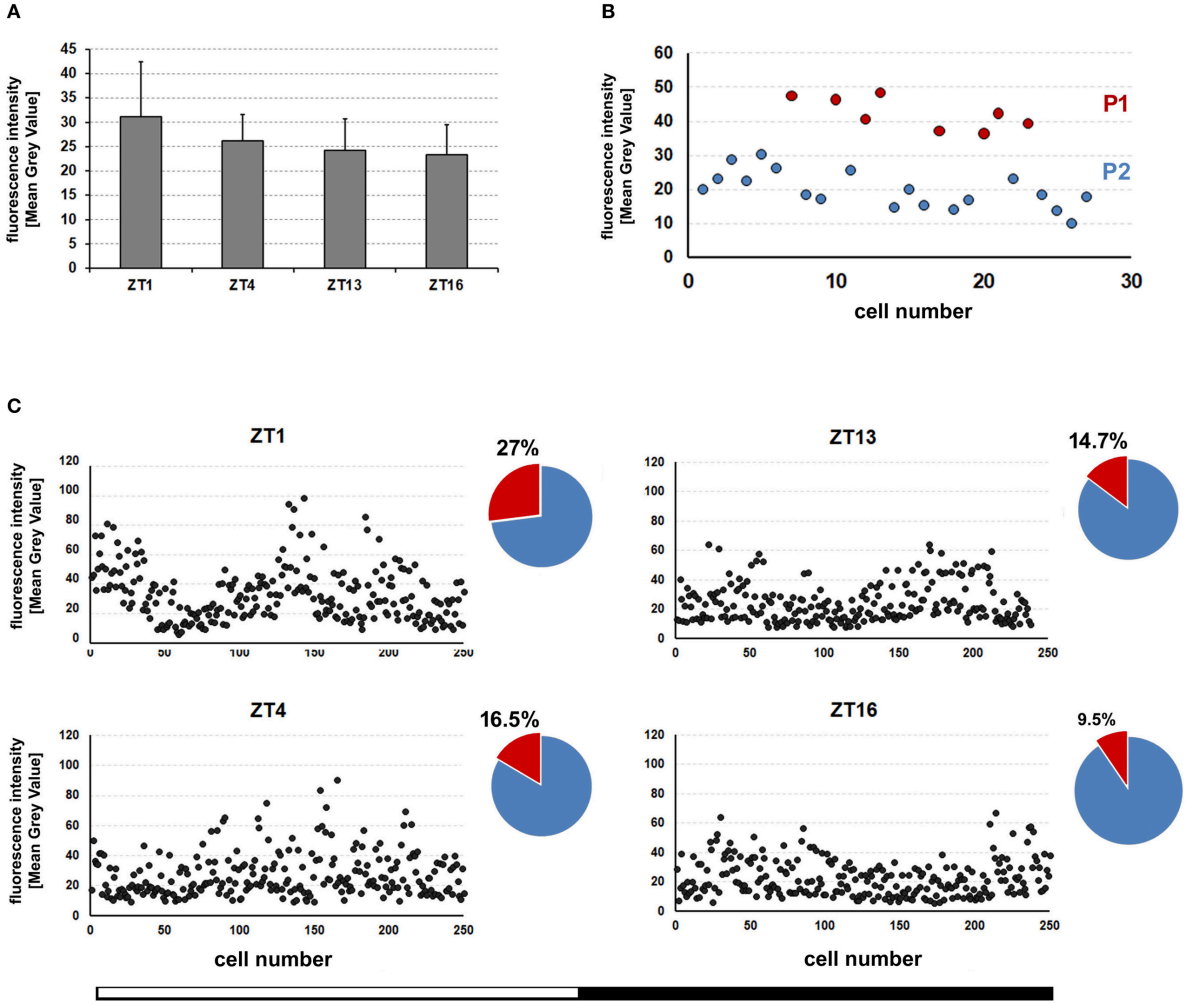
**(A)** The average (±*SD*) level of REPO-specific fluorescence in the nuclei of the dMnGl of CS flies collected at different times (ZT1, ZT4, ZT13, and ZT16) of the day. For each time point the fluorescence was measured in the dMnGl of 10 hemispheres (*N* = 10). **(B)** The consecutive measurements of REPO fluorescence in the dMnGl (*N* = 27) of an exemplary optic lobe, revealing distinct populations of cells with higher (P1) and lower (P2) levels of fluorescence at ZT1. **(C)** The measurements of REPO fluorescence in the dMnGl of flies collected at designated ZTs. Each chart shows measurements for 250 nuclei from 10 optic lobes. The accompanying pie charts illustrate percentages of nuclei with the intensity of fluorescence below (blue) and above (red) the MGV of 40.

Next, we wanted to know whether the level of REPO in the dMnGl changes during the day, which would imply daily oscillations in the REPO-controlled glial functions. It turned out that the average level of REPO-specific immunofluorescence measured in all the dMnGl at different times (ZTs) of the day and night (Figure 3A) did not show statistically significant differences (Kruskal-Wallis Test: *H*[*df* = 3, *N* = 40] = 2.8, *p* > 0.05). On the other hand, based on the Mean Gray Value (MGV) individual cells were distributed in two groups that can be identified as P1 and P2 (Figure 3B). Closer inspection revealed that the percentage of P1 (MGV > 40) and P2 (MGV < 40) vary throughout the day (as shown in Figure 3C). The number of P1 cells is bigger during the day than at night as shown for the population of 250 cells (from 10 specimens).

### ALGl express PER and REPO at low levels

**Figure 4:**
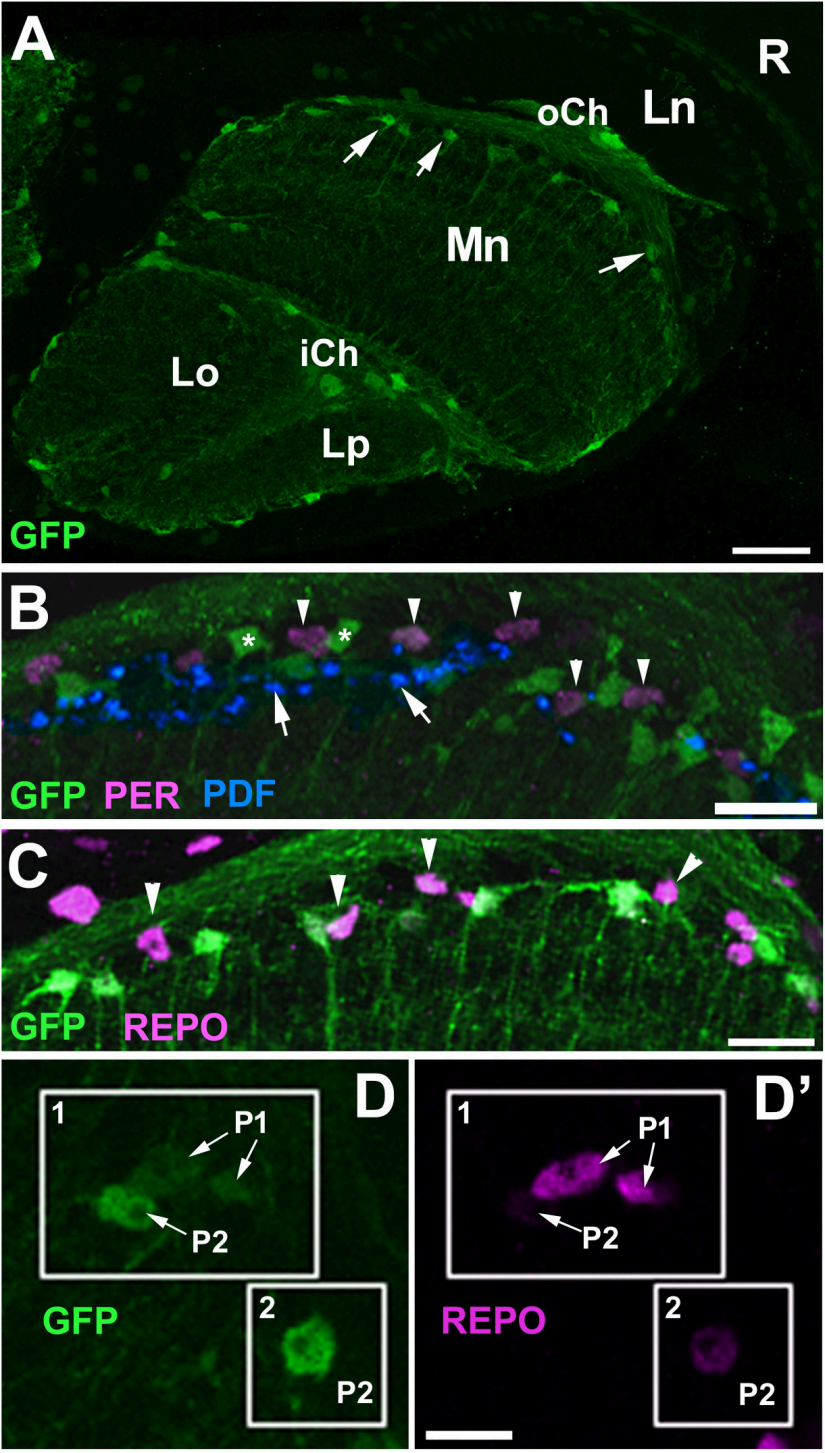
Colocalization of PER and REPO to the astrocyte-like glia (ALGl) of *alrm*-Gal4/UAS-S65T-GFP flies collected at ZT1. **(A)** Localization of ALGl (arrows) in the distal part of the medulla neuropil (Mn) in horizontal section. R, retina; Ln, lamina neuropil; oCh, outer chiasm; iCh, inner chiasm; Lo, lobula; Lp, lobula plate. Scale bar: 20 μm. **(B)** GFP-expressing ALGl (asterisks) in the region of PDF-positive terminals (arrows) of l-LNv neurons do not express PER. PER-positive nuclei (arrowheads) are located in between the cell bodies of ALGl. Scale bar: 10 μm. **(C)** GFP-negative nuclei of non-ALGl (arrowheads) express REPO at high level. Scale bar: 10 μm. (**D**,**D'**) ALGl and non-ALGl cells of the distal medulla in higher magnification. ALGl cells (box 1 and 2) that express GFP at high level **(D)**, show weak expression of REPO **(D')**, whereas non-ALGl cells (box 1) that express GFP at barely detectable level **(D)**, show strong REPO expression **(D')**. Scale bar: 5 μm.

Then, we wanted to find out whether the ALGl and EnGl can be associated with the two categories of cells: P1 and P2. In order to do this we determined the relative levels of PER and REPO expression in these two types of glia. Initially, we performed anti-PER and anti-REPO immunolabeling on the tissue of *alrm*-Gal4/UAS-S65T-GFP transgenic flies. The *alrm*-Gal4 driver is known to be specific for the ALGl cells and so S65T-GFP can be observed in their cytoplasm and nuclei (Figure 4A). The immunolabeling did not reveal any presence of PER in the GFP positive cells (ALGl), suggesting that these cells either do not express PER at all, or express it at non-detectable levels (Figure 4B). We found, however, that the ALGl express REPO at the level characteristic for the P2 cells (Figures 4C,D,D'). The ALGl cells do not belong, therefore, to the P1 population of either PER- or REPO-positive cells. They can be classified as P2 with respect to REPO expression. Whether these cells do express small amounts of PER could not be determined based on this staining. Interestingly, the REPO-P1 cells display a very weak expression of GFP reporter of *alrm*-Gal4 driver (Figures 4D,D').

**Figure 5:**
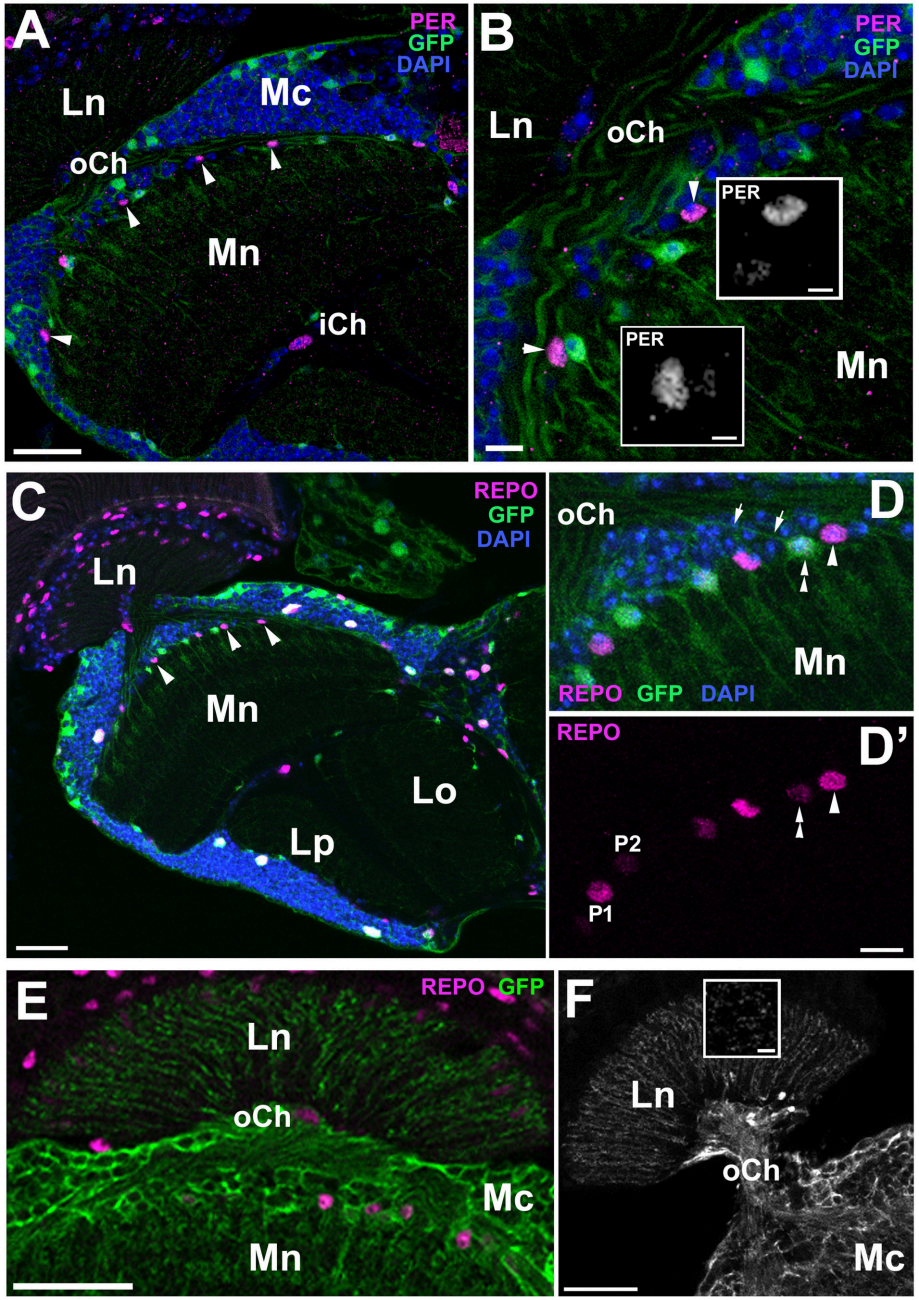
Frontal sections of the optic lobe of *EAAT1*-Gal4/UAS-S65T-GFP and *EAAT1*-Gal4/UAS-mCD8-GFP transgenic flies collected at ZT1 and immunolabeled with anti-PER and anti-REPO antibodies. **(A)** PER-positive nuclei of the dMnGl (arrowheads) are clearly visible in the distal part of the medulla neuropil (Mn) of *EAAT1*-Gal4/UAS-S65T-GFP flies. Ln, lamina neuropil; oCh, outer chiasm; Mc, medulla cortex; iCh, inner chiasm. Scale bar: 20 μm. **(B)** Higher magnification of the distal medulla showing that GFP-positive cells express PER (inserts) at barely detectable level. The neighboring GFP-negative cells (arrowheads) express PER at high level. Scale bar: 5 μm and 2 μm (in inserts). **(C)** REPO-positive nuclei of the dMnGl (arrowheads) are well visible in the distal part of the medulla neuropil (Mn) of *EAAT1*-Gal4/UAS-S65T-GFP flies. Ln, lamina neuropil; Lo, lobula; Lp, lobula plate. Scale bar: 20 μm. **(D,D')** Higher magnification of the distal medulla neuropil (Mn) of *EAAT1*-Gal4/UAS-S65T-GFP flies viewed in a projection of 10 μm thick image stack. GFP-negative cells (arrowhead in **D**) express REPO at higher level (arrowhead in **D'**), whereas GFP-positive cells (double arrowhead in **D**) express lower level of REPO (double arrowhead in **D'**). Some of GFP-positive cells send projections to the lamina (arrows in **D**). DAPI-positive nuclei visualize the medulla cortex. oCh, outer chiasm. Scale bar: 5 μm. **(E,F)** Morphology of GFP-positive processes in the lamina of *EAAT1*-Gal4/UAS-mCD8-GFP flies in frontal **(E)** and horizontal **(F)** sections. The basket-like terminals, which surround each of the lamina synaptic units, so called cartridges, are characteristic for the processes of T1 interneurons. Insert in **(F)** shows the cross section of T1 processes in the lamina. Ln, lamina neuropil; oCh, outer chiasm; Mc, medulla cortex; Mn, medulla neuropil. Scale bars: 20 μm for **(E,F)**, 5 μm for insert.

The issues outlined above were subsequently resolved using the GFP reporter of a driver for the *Drosophila Excitatory Amino Acid Transporter 1, EAAT1*-Gal4, which also marks the glia that populate the distal medulla neuropil (Edwards et al., 2012). The obtained immunolabeling of *EAAT1*-Gal4/UAS-S65T-GFP optic lobes confirmed that the levels of expression of PER and REPO in the GFP-positive cells (the ALGl nuclei) are lower than in the P1 cells (Figures 5A,B,D,D'). PER is detectable, but its level is exceptionally low (inserts in Figure 5B). On this view, the GFP-positive cells (ALGl) should be regarded as P2 with respect to both REPO and PER expressions.

We have noticed, however, that the P2 population of cells does not consist entirely of glia. At least some of these GFP-positive cells send projections to the first optic neuropil-the lamina (Figure 5D), which means that they do not belong to the ALGl. Their terminals in the lamina (Figures 5E,F), as revealed by the driver based expression of membranous mCD8-GFP reporter (*EAAT1*-Gal4/UAS-mCD8-GFP), have the morphology of baskets enveloping the lamina synaptic units, called cartridges (insert in Figure 5F). Since such a morphology is typical for T1 interneurons, we assume that *EAAT1*-Gal4 drives expression also to T1 cells, and that they belong to the P2 group.

### EnGl express high levels of PER and REPO

**Figure 6:**
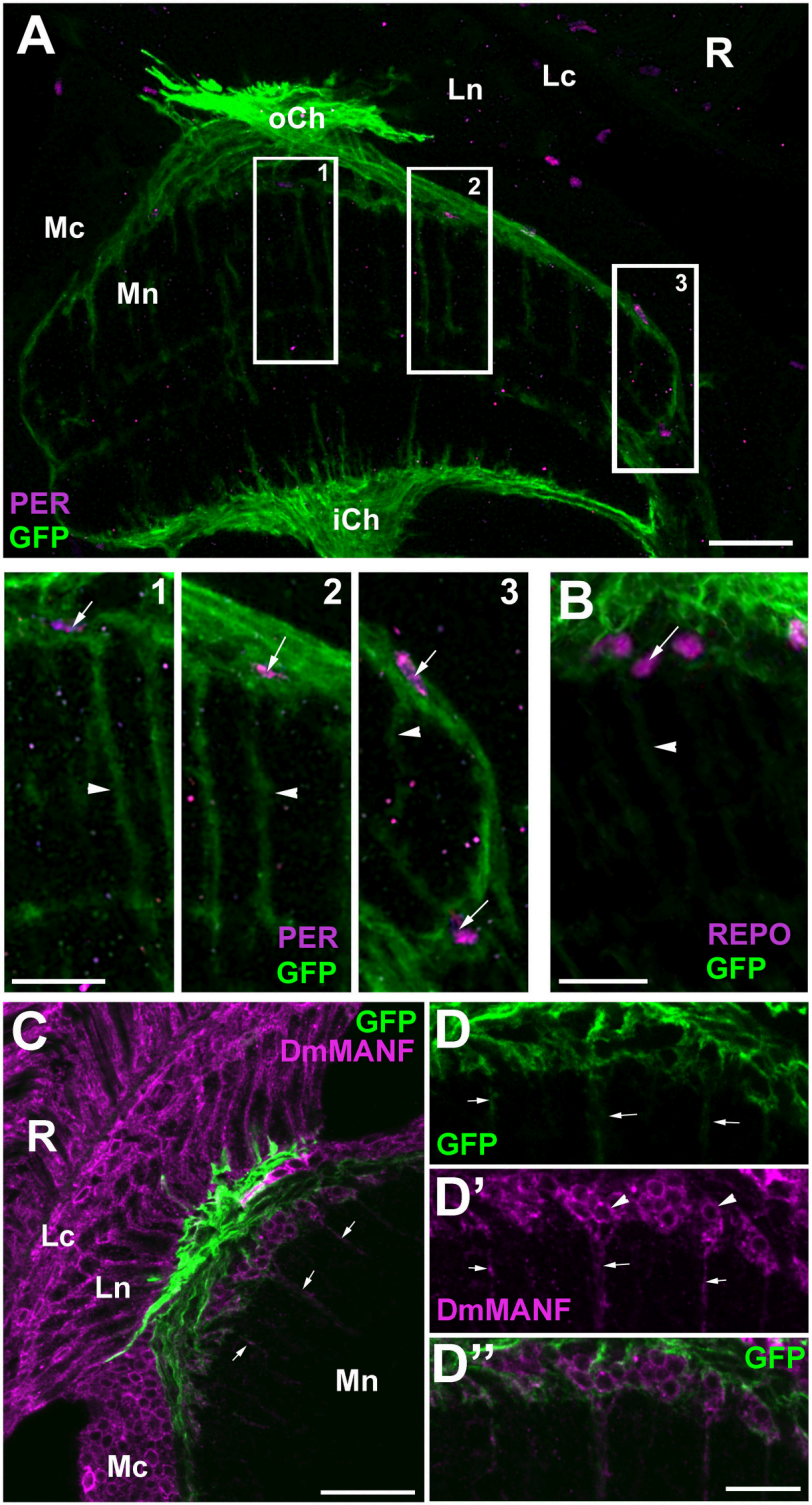
**(A)** Horizontal section of the medulla of *NP6520*-Gal4/UAS-mCD8-GFP flies immunolabeled with anti-PER antibody. The nuclei of GFP-expressing EnGl (frames 1–3) are PER-positive, which is well visible in higher magnification (panels 1–3). The processes of EnGl (arrowheads in panels 1–3) span the medula neuropil (Mn). R, retina; Lc, Lamina cortex; Ln, lamina neuropil; oCh, outer chiasm; Mc, medulla cortex; iCh, inner chiasm. Scale bars: 20 μm for **(A)** and 10 μm for panel 1, 2, and 3. **(B)** The nuclei of GFP-positive EnGl show high level of REPO-specific immunofluorescence (arrow). Arrowhead-the EnGl processes. Scale bar: 10 μm. **(C)** The distal part of the medulla in horizontal section of *NP6520*-Gal4/UAS-mCD8-GFP flies immunolabeled with anti-DmMANF antibody. The processes of EnGl are marked with arrows. R, retina; Lc, lamina cortex; Ln, lamina neuropil; Mc, medulla cortex; Mn, medulla neuropil. Scale bar: 20 μm. **(D-D”)** Higher magnification of EnGl marked in **(C)**. The GFP-positive processes of EnGl (**D**, arrows) are marked with DmMANF **(D',D”)**. DmMANF is also visible in the perinuclear space of cell bodies in the medulla cortex (D', arrowheads). Scale bar: 10 μm.

To confirm that it is the EnGl that express PER and REPO at high levels we performed anti-PER and anti-REPO immunolabeling on the tissue of *NP6520*-Gal4/UAS-mCD8-GFP transgenic flies. *NP6520*-Gal4 is the marker for ensheathing glia (Awasaki et al., 2008; Richier et al., 2017). The immunolabeling revealed strong expression of PER and REPO in GFP-positive cells (EnGl) at the border of cortex and neuropil of the medulla (Figure 6). The EnGl of the distal medulla express also *Drosophila melanogaster* MESENCEPHALIC ASTROCYTE-DERIVED NEUROTROPHIC FACTOR (DmMANF), as revealed by the immunolabeling using anti-DmMANF Ab (Figure 6C). It is present not only in the perinuclear space of endoplasmic reticulum (ER) of EnGl, but also in their long processes that penetrate the neuropil of the medulla (Figures 6C–D”). In fact, DmMANF was mostly observed in the processes of EnGl in the medulla neuropil, as the majority of DmMANF-positive processes that were most clearly visible belonged to EnGl (were also GFP-positive; Figures 6D–D”).

### Both P1 and P2 cells express TIM

**Figure 7:**
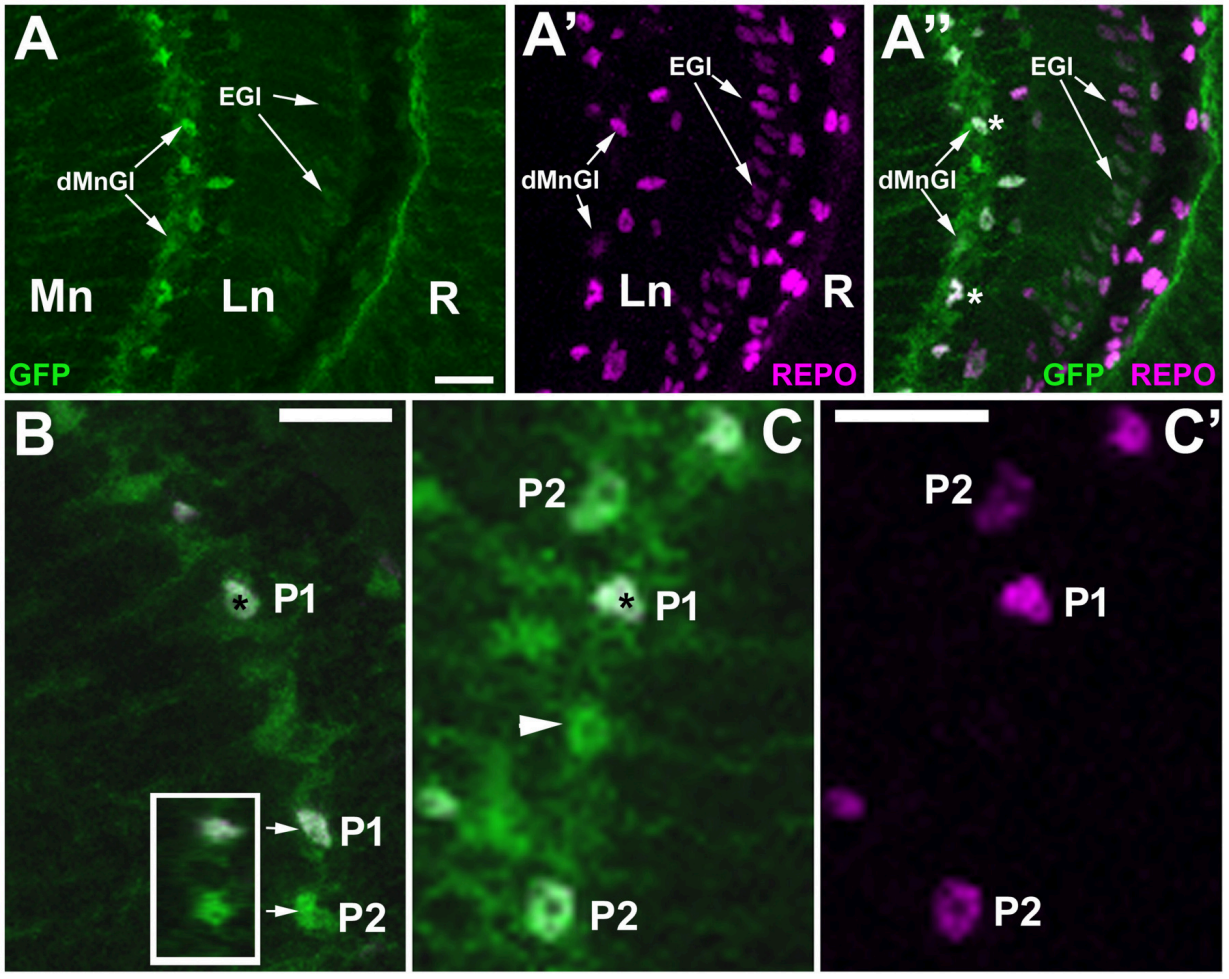
Anti-REPO immunolabeling of GFP-labeled *tim*-expressing cells in the visual system of *tim*-Gal4/UAS-S65T-GFP flies collected at ZT1. **(A–A”)** The strong fluorescence of GFP-reporter is observed in the dMnGl, whereas the weak fluorescence is visible in the other glial cells, e.g., the epithelial glial cells (EGl) of the lamina neuropil (Ln). R, retina; Mn, medulla neuropil. **(B)** Exemplary GFP-positive cells revealing higher (P1) and lower (P2) levels of REPO-specific fluorescence. Insert: the orthogonal view (*z*-stack projection) of P1 and P2 (arrows) showing that their nuclei are complete (so the difference in the intensity of REPO-specific immunofluorescence results entirely from the differences in the level of REPO expression). **(C,C')** TIM-positive cells (green) in the distal medulla show either high (P1), low (P2), or no expression of REPO (arrowhead). The nuclei of cells revealing high level of expression of GFP and REPO are marked with asterisks. Scale bars in all panels: 10 μm.

*tim*-gal4 line is often used as a driver line for clock cells (Ozkaya and Rosato, 2012) due to its strong expression in pacemakers. Since our other study (Górska-Andrzejak et al., 2018) revealed that the dMnGl express GFP reporter of *tim-*Gal4 at much higher level than the rest of the optic lobe (Figure 7A), we expected TIM to be expressed at high level in the P1 cells (Figure 7). Our results showed, however, that not only the P1 but also the P2 and some of the REPO-negative cells express TIM (Figure 7C). It is worth emphasizing that we did not see any negative correspondence of REPO level with the GFP reporter of tim, in contrast to what we observed for *alrm*-GFP and *EAAT1*-GFP lines (Figures 4, 5).

## Discussion

### The dMnGl express different levels of PER and REPO

Our detailed microscopic investigation revealed that the dMnGl, which are known to contain two morphologically distinct types of glia, the ALGl, and EnGl (Awasaki et al., 2008; Doherty et al., 2009; Edwards and Meinertzhagen, 2010; Hartline, 2011; Edwards et al., 2012), differ with respect to the level of expression of the clock protein PER and the glial marker REPO. Surprisingly, the ALGl express low levels of both PER and REPO (P2), whereas the EnGl display relatively high levels of these two proteins (P1). The amplitude of the circadian (PER-controlled) and glial (REPO-controlled) functions of ALGl and EnGl may therefore, significantly differ.

Obviously, the mere findings that different cells express different levels of PER do not necessarily imply that their molecular clocks are substantially different, especially that the clock proteins may be also engaged in non-clock functions (Beaver et al., 2003; Sakai et al., 2004; Houl et al., 2006; Chen et al., 2012). For example, the fruit fly's *per* (and none of the other clock genes) is crucial also for the formation of long-term memory (LTM). *per* expressing cells that regulate the LTM formation might, therefore, be distinct from the clock neurons (Sakai et al., 2004; Chen et al., 2012). The fact that such difference between ALGl and EnGl is not visible in the case of TIM expression (based on the level of GFP reporter of *tim-*Gal4 driver; Figure 7) could also indicate an additional function of PER (next to the clock function) in either both or only one of these two types of dMnGl glia.

Nevertheless, it is also known that the abundance of PER, the prime repressor in the mechanism of the circadian clock (Landskron et al., 2009; Hardin, 2011), sets the pace of the clock and the phase of the circadian rhythm (Baylies et al., 1987). Levels of *per* RNA are correlated with the period length. Therefore, flies with the lowest levels of PER have slow-running biological clocks (Baylies et al., 1987) and the phase of the rhythm can be altered by temporary increase of PER concentration (Edery et al., 1994). Hence, in the cells that express PER at different levels, such as P1 and P2, the pace of the clocks may differ. Additionally, as we saw in our other study (Górska-Andrzejak et al., 2018), the dMnGl expressed the highest level of GFP of all the optic lobe glia, when examined in *tim*-Gal4/UAS-S65T-GFP transgenic flies. Consequently, at least some of the dMnGl must have high levels of both TIM and PER, and so may belong to the robust molecular oscillators (with high-amplitude cycling of both PER and TIM) among the glia. The fact that the percentage of dMnGl with the highest level of REPO displays daily fluctuations supports this notion, especially because the timing of its maximum (at ZT1) is consistent with an increased PDF immunoreactivity seen early in the morning (Park et al., 2000).

### The ALGl reveal low levels of PER and REPO

As we were unable to colocalize the *alrm-*Gal4 and *EAAT1-*Gal4-driven GFP with high levels of PER and REPO we conclude that the astrocytic glia belong to the P2 population of dMnGl. No staining of PER in the ALGl was also observed by Long and Giebultowicz (2018) in several areas of the central brain at two different time points (ZT22 and ZT10), which typically correspond with high and low PER expression. It is thus unlikely that low level of PER at ZT1 can be caused by some untypical daily pattern of PER expression. Our study of dMnGl conducted at several time points (Górska-Andrzejak et al., 2018) did not reveal any irregularities in the daily pattern of PER that would indicate untypical expression in the ALGl. On this view, we are quite confident that the ALGl should be classified as the P2 cells.

The low level of PER in the ALGl of *Drosophila* is surprising since these glia were reported to be crucial in behavioral rhythmicity (Suh and Jackson, 2007; Ng et al., 2011). Surprising as it may be, the well-documented role of ALGl in behavioral rhythmicity indicates that also the cells, like ALGl, that express PER at low (or not detectable) levels may play important roles in the circadian network of *Drosophila*. It is worth mentioning, that the majority of ALGl express the glia-specific protein, EBONY (Jackson et al., 2015), which cycles in the fly's head under control of PER/TIM-based intracellular oscillator (Ueda et al., 2002; Suh and Jackson, 2007), and which is vital in regulation of behavioral rhythmicity (Suh and Jackson, 2007). The *ebony* gene mutation was shown to alter the circadian activity rhythms (Newby and Jackson, 1991) and its phenotype can be rescued by induction of *ebony* expression under *repo*-Gal4 driver (Suh and Jackson, 2007).

Perhaps the glia that express EBONY (or other proteins similar to EBONY) need not express PER at such a high level to play the function of the peripheral clocks. Incidentally, another group of the neuropil glia that express EBONY are the epithelial glial cells (EGl; Górska-Andrzejak et al., 2009) of the lamina (Figure 1A), which like ALGl of the distal medulla express relatively low levels of PER (Figure 1B) and REPO (Figures 2A,D). Being regarded as the astrocyte-type glia of the lamina, in spite of their columnar rather than astrocytic morphology (Edwards and Meinertzhagen, 2010), they are another example of the ALGl that display low levels of PER and REPO.

### It is the EnGl that express higher levels of PER and REPO

Our study have revealed that the EnGl express PER at high level. Therefore, they may be regarded as robust oscillators in comparison with other types of glia, just like the l-LNv neurons in comparison with other clock neurons (Helfrich-Förster, 1998; Helfrich-Förster et al., 2007). The l-LNvs display the molecular rhythms of PER and TIM that are phase-advanced and of higher amplitude with respect to other clock neurons. Therefore, it is presumed that their input may be particularly robust and they are vital for light-mediated modulation of arousal and sleep (Sheeba et al., 2008, 2010). The question remains whether the EnGl support l-LNvs in this modulation. In this context, the fact that the percentage of REPO-P1 cells was found to be the biggest at the beginning of the day (Figure 3) also seems to be relevant.

It is an interesting coincidence that the age-related decline in PER occurred in various glial subtypes, but was not observed in the case of EnGl (Long and Giebultowicz, 2018; study on the age-dependent changes in the expression of PER in the *Drosophila* glia). This confirms their robustness as glial oscillators. The age related decline in PER does not occur in the neuronal pacemakers of the central clock, but it has been found in the photoreceptors of the retina (Long and Giebultowicz, 2018).

The higher level of PER (and REPO) in EnGl may indicate the need for stronger circadian regulation of the functions carried out by EnGl, e.g., the ensheathment of R-cells axon bundles. Being the robust circadian oscillators, the EnGl could modulate their capacity to process information in a circadian manner. As they wrap and insulate neuronal axons (Cameron et al., 2016), they increase the conduction velocity of electrical impulses (in both vertebrate and invertebrate nervous systems), and consequently, enhance the neuronal capacity to process information at a faster pace.

Studies on development of the nervous system in *Drosophila* indicate that this type of glia are able to undergo complex morphological changes to accommodate neuronal axons (Banerjee and Bhat, 2008; Subramanian et al., 2017). In the adult brain, on the other hand, the ensheathing glia (but not astrocytes) express the engulfment receptor *Draper*, which enables glial membranes to contact degenerating axons and proceed with engulfment of axonal debris. Blocking of endocytosis specifically in the EnGl cells inhibits the process of severed axons clearance (Doherty et al., 2009).

Interestingly, as we showed the EnGl of *Drosophila* express the DmMANF, a novel evolutionary conserved (in ~50% identical to human MANF) neurotrophic factor (Petrova et al., 2003) that not only protects neurons against apoptosis and supports their survival (like other neurotrophic factors), but also plays a conserved immune modulatory function (Neves et al., 2016). It has been shown that the silencing of DmMANF in glial cells induces the appearance of so called MANF immunoreactive cells (MiCs) during metamorphosis. These are the migratory cells that closely resemble macrophages/hemocytes and vertebrate microglia. They express RELISH and DRAPER suggestive of their immune response activation (Stratoulias and Heino, 2015b).

Thus, the ensheathing glia appear to play the similar role as the highly rhythmic microglia in the mammalian brain (for review see Salter and Stevens, 2017). For example, the hippocampal microglia have been shown to rhythmically expresses core clock genes: *Per1, Per2, Rev-erb, Bmal1*, as well as several pro-inflammatory cytokines. They also display profound differences in immune stimulation throughout the day (Hayashi et al., 2013; Fonken et al., 2015). The strong molecular rhythm of PER oscillations in the *Drosophila* EnGl definitely adds to its similarity to microglia.

In the *Drosophila* embryonic nervous system DmMANF can be found only in glia (Palgi et al., 2009), while it is present in both the glia and the neurons of the adult brain (Stratoulias and Heino, 2015a). In glia it is present in both cell somata and processes. In neurons, on the other hand, it can be found only in the somata (Stratoulias and Heino, 2015a). In our recent study (Walkowicz et al., 2017) we found that DmMANF is strongly expressed in the epithelial glial cells (EGl) of the first visual neuropil (the lamina). Here we show, that it is also clearly present in the cell bodies and, most importantly, the processes of EnGl (Figures 6C,D”).

The processes of EnGl in the distal medulla are enlarged and form a broad meshwork (Kremer et al., 2017). Since they are closely associated with R7 and R8 photoreceptor terminals, just like the processes of the lamina ensheathing glia (also called marginal glia) with the terminals of R1-R6 photoreceptors (Edwards et al., 2012; Kremer et al., 2017), one might put forth a hypothesis that the EnGl may be involved in the regulation of daily morphological changes in the photoreceptor terminals in the distal medulla. In the *Drosophila* medulla various types of axons innervate the neuropil unit (column), but they must target distinct layers while using the same tract. Even the subtle structural changes provided by the EnGl should thus enable modulation of the information flow during the day and night, affecting functions of the visual system. In the mammalian retina renewal of the photoreceptor outer segments is regulated in a circadian manner and this process is based on engulfing and phagocytosis of photoreceptors by the neighboring pigmented epithelium (reviewed in Kevany and Palczewski, 2010).

We found that the EnGl express REPO at higher level than the ALGl. It seems important in view of the recent findings (Matsuno et al., 2015) that the expression of REPO (regulated by neuronal protein KLINGON, KLG), just like *per* expression (Sakai et al., 2004; Chen et al., 2012), is vital for the formation of the long term memory (LTM). Experiments showed that *repo* mutants are defective for LTM and conditional knockdown of *repo* under control of ALGl-specific driver line results in reduction of 24 h memory, while it is not affected by similar inhibition in EnGl (Matsuno et al., 2015). It appears, therefore, that only the astrocyte glia is involved in this process. One should take into consideration, however, that this may be also caused by the fact that the EnGl, even without REPO, should still be able to partake in LTM formation, owing to the high level of PER, exceeding that in the ALGl.

The capability of changing morphology and governing the phagocytosis, as well as for the high expression of PER and daily fluctuations in REPO abundance suggest that both morphology and functioning of the EnGl of distal medulla has the capacity to undergo robust daily/circadian changes. The occurrence of daily alterations in glial morphology is known from the study on the epithelial glia of the lamina in the housefly (Pyza and Górska-Andrzejak, 2004). They are associated with oscillations of the axon diameter and the size of dendritic tree in L2 monopolar cells during the day and night (Pyza and Meinertzhagen, 1999; Weber et al., 2009). Moreover, the contribution of glial clocks to the circadian structural plasticity of the pacemaker neurons (the small LNvs) have just been reported by Herrero et al. (2017). The impaired glial clocks abolish circadian structural remodeling without affecting other clock-controlled outputs (Herrero et al., 2017).

### The T1 interneurons express PER and REPO at very low levels

Interestingly, the so called T1 interneurons (Hamanaka and Meinertzhagen, 2010) first identified by Fischbach and Dittrich (1989) occurred to display low levels of PER and REPO. Because of the very presence of PER, we cannot rule out the possibility that the T1 interneurons are part of the medulla circadian network. Interestingly, however, these cells are perceived as neuronal anomaly (Takemura et al., 2008) as they fail to make presynaptic sites at chemical synapses in both the lamina and the medulla neuropils (that they innervate). Since their outputs have not been found in detailed EM study, the cells appear to be entirely postsynaptic (Hamanaka and Meinertzhagen, 2010). What we have found about T1 makes this cell even more peculiar (but also more interesting) as it expresses a very low level of the glial marker, REPO (Figure 5), in addition to expression of EAAT1 transporter (also often expressed by glia) that mediates the high affinity uptake of glutamate or aspartate into cells. Unveiling its precise function, however, requires further study.

## Conclusions

Our results revealed that the two types of dMnGl, the astrocyte like glia (ALGl) and the ensheathing glia (EnGl), differ among each other also with respect to the level of expression of the clock protein PER and the glial marker REPO. Since the ALGl glia controlling circadian behavior show little or no PER expression we conclude that the high levels of PER may not be necessary for the circadian functions in the peripheral oscillators, such as the ALGl. This makes the functioning of the circadian mechanism in the ALGl and other peripheral oscillators even more intriguing. On the other hand, the high level of PER and REPO in the EnGl, as well as the presence of daily changes in the number of cells that express REPO at relatively high levels, may indicate that the EnGl are highly rhythmic, just like their vertebrate counterparts, the microglia. They may constitute the particularly important and/or influential glial component of the *Drosophila* circadian network and deserve more consideration in future study on the role of glia in the circadian rhythms.

Our study reveal the heterogeneity of glial oscillators and the complexity of the distal medulla circadian network, thus establishing an essential basis for the study of its functioning. We believe that further study should be focused on the type-dependent properties of glia in the circadian network and clarification of the EnGl circadian function.

## Author contributions

WK and LW: performed experiments, collected and analyzed data, and prepared the manuscript. AP: performed some experiments, collected, and analyzed data. JG-A: designed the study, performed experiments, collected and analyzed data, and prepared the manuscript.

### Conflict of interest statement

The authors declare that the research was conducted in the absence of any commercial or financial relationships that could be construed as a potential conflict of interest.
